# Affimer reagents as tools in diagnosing plant virus diseases

**DOI:** 10.1038/s41598-019-43945-6

**Published:** 2019-05-17

**Authors:** Emma L. Hesketh, Christian Tiede, Hope Adamson, Thomas L. Adams, Matthew J. Byrne, Yulia Meshcheriakova, Inga Kruse, Michael J. McPherson, George P. Lomonossoff, Darren C. Tomlinson, Neil A. Ranson

**Affiliations:** 1 Astbury Centre for Structural Molecular Biology, School of Molecular and Cellular Biology, Faculty of Biological Sciences, University of Leeds, Leeds, LS2 9JT UK; 2 School of Biomedical Sciences, Faculty of Biological Sciences, University of Leeds, Leeds, LS2 9JT UK; 3 Department of Biological Chemistry, John Innes Centre, Norwich Research Park, Colney, Norwich NR4 7UH UK; 4 Present Address: Strathclyde Institute of Pharmacy & Biomedical Sciences, 161 Cathedral St, Glasgow, G4 0RE UK

**Keywords:** Assay systems, Cryoelectron microscopy

## Abstract

Plant viruses can cause devastating losses to agriculture and are therefore a major threat to food security. The rapid identification of virally-infected crops allowing containment is essential to limit such threats, but plant viral diseases can be extremely challenging to diagnose. An ideal method for plant virus diagnosis would be a device which can be implemented easily in the field. Such devices require a binding reagent that is specific for the virus of interest. We chose to investigate the use of Affimer reagents, artificial binding proteins and a model plant virus Cowpea Mosaic virus (CPMV) empty virus like particles (eVLPs). CPMV-eVLP mimic the morphology of wild-type (WT) CPMV but lack any infectious genomic material and so do not have biocontainment issues. We have produced and purified an Affimer reagent selected for its ability to bind to CPMV-eVLP and have shown that the selected Affimer also specifically binds to WT CPMV. We have produced a 3.4 Å structure of WT CPMV bound to the Affimer using cryo-electron microscopy. Finally, we have shown that this Affimer is capable of reliably detecting the virus in crude extracts of CPMV-infected leaves and can therefore form the basis for the future development of diagnostic tests.

## Introduction

Food security is a global challenge; over 6% of global food production is lost due to pathogens including viruses^1^. In the field, plant virus infections are often unmanaged or managed incorrectly owing to difficulties in diagnosis. Rapid diagnosis of a plant viral infection and therefore effective containment of the infection is required to minimize the spread of disease to other plants and limit crop losses. Unfortunately, for many plant viruses this is not practical as the methods of detection are either serology which depends on a specific antibodies (many of which are not available) or nucleic acid extraction, amplification and genome sequencing. Both of these techniques require transporting samples to a distant laboratory, adding time and expense to diagnosis. Consequently, there is a requirement for more efficient, quick and cheap tests that are capable of reliably identifying plant virus infections.

Antibodies (where available) have been used for detection of plant viral infections^2^. However, there are a number of limitations associated with their use as in-field diagnostics. The generation of an antibody generally requires the availability of significant quantities of the purified target virus, and this can be difficult or impossible to obtain. Antibodies are also expensive to generate and manufacture, and they can experience significant stability issues in the relatively harsh environments in which an in-field diagnostic test would be used. For this reason, we chose to explore the use of Affimer reagents as an alternative, non-antibody binding reagent^3,4^. Affimer proteins are novel affinity reagents which mimic the molecular recognition of antibodies, but can be isolated quickly and produced recombinantly. They are synthetic and are based on either a human cystatin A (type I) or a plant-derived phytocystatin consensus scaffold (type II). Affimer proteins are small (11 kDa, 3 nm in diameter), stable (70 °C < T_m_ < over 100 °C), monomeric proteins that lack disulphide bonds^3^. Their structure consists of a single α-helix and four β-strands, with the molecular recognition sites being located within two variable loops, each up to nine amino acids in length^3^. Their advantages include stability and their relatively inexpensive, simple production. Large phage display libraries (~1.3 × 10^10^ variants) have been generated^3,4^ and screened against hundreds of target proteins. A wide range of Affimer binders have now been identified from these phage libraries^4^ to a wide range of targets and have been successfully used for applications as diverse as molecular research tools^4–9^, biosensors^6^ and immunodiagnostics^7^. Importantly, Affimer proteins have been shown to be highly specific, and thus able to distinguish between closely-related targets^3–5,8^.

In addition to the challenges of using an antibody as a diagnostic reagent, there are also significant problems with using wild-type (WT) infectious viruses to produce those antibodies (or any other binding reagent). Perhaps unsurprisingly, there is a lack of correlation between the pathogenicity of a virus and the level to which it accumulates in the tissues of infected plants (for example ref.^9^). Thus, many economically important and highly pathogenic plant viruses accumulate to only low titres in infected plants before killing them. There are also very significant biocontainment issues associated with producing the large amounts of an infectious entity that are typically required for antibody production. Virus-like particles (VLPs) can potentially be produced more easily and to a much higher concentration than the parent virus, and because VLPs are produced in the absence of the viral genome, and thus cannot be infectious, they are a fundamentally safer alternative to the use of WT virus. As a result, in recent years VLPs have been exploited in biotechnology for antibody production and many other applications^10,11^.

Here we sought to investigate whether VLPs could be used to generate an Affimer reagent that would be cross-reactive to a WT virus. For this analysis we used Cowpea Mosaic Virus (CPMV), a plant virus from the order *Picornavirales* that has been used extensively in biotechnology and as a model for single-stranded RNA viruses more generally. This is an ideal model system as empty virus-like particles (eVLPs) of CPMV are readily produced and purified^12^ using a well-established expression system. In addition, there is a large amount of structural information for both WT CPMV and CPMV eVLPs^13–16^. Using CPMV eVLPs we have identified an anti-eVLP Affimer and characterized its affinity for WT, infectious CPMV. We also determined the structure of CPMV bound to the selected Affimer using cryo-electron microscopy (cryo-EM). Finally, we demonstrate that the selected Affimer can detect the presence of CPMV directly within crude extracts of infected leaves, showing that the eVLP/Affimer combination is a potential route to the development of new in-field diagnostics.

## Results and Discussion

### WT CPMV and eVLP have the same antigenicity

**Figure 1:**
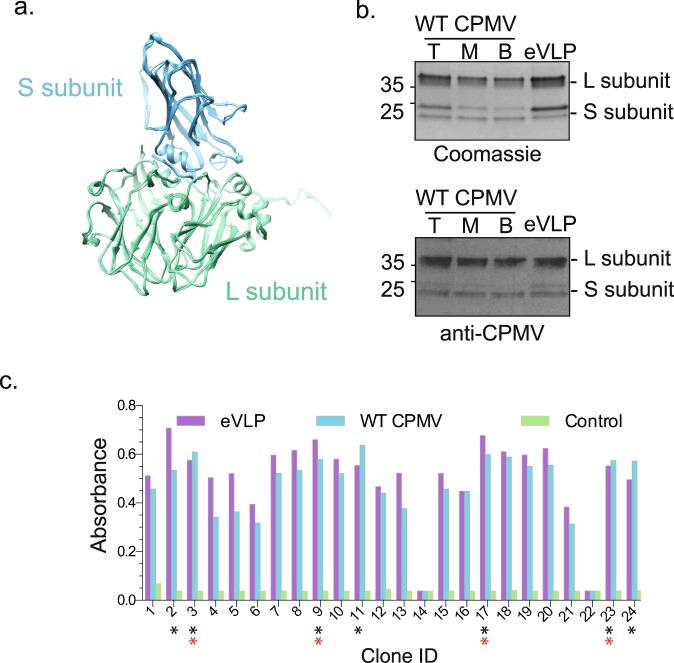
Wild type CPMV and CPMV eVLP have the same antigenicity. (**a**) Overlay of published CPMV structures, 5a33, 5a32, 5FMO, 5MS1, 5MSH. The RMSD between these structures is 0.4. The Small (S) subunit is coloured blue and the Large (L) subunit is coloured green. (**b**) Coomassie blue-stained SDS-PAGE gel to show the total protein concentration of CPMV samples compared with a western blot which demonstrates that a polyclonal antiserum raised against WT CPMV detects both the L and S subunits of WT CPMV-T, M and B and eVLP-CPMV with the same efficiency. (**c**) Phage ELISA of Affimer proteins from 24 clones incubated in wells containing immobilised empty virus like particles (eVLPs) (pink), WT CPMV (blue) and a negative control (green), showing the 3,3′,5,5′-tetramethylbenzidine (TMB) product absorbance at 620 nm after 2 minutes. Clones labelled with a black asterisk (*) were selected as Affimer proteins suitable for detecting both eVLP and WT CPMV and were sequenced. Clones labelled with a red asterisk were identified as Affimer proteins with different sequences at the variable loops and used for further testing.

CPMV is an icosahedral virus composed of 60 copies of both the Large (L) and the Small (S) coat protein subunit^16^. CPMV has a bipartite ssRNA genome (RNA-1, 6 kb and RNA-2, 3.5 kb). Each of the two genomic segments is encapsidated separately producing three fractions named for their sedimentation behavior in density gradients: CPMV-B(ottom) (containing the larger RNA-1), CPMV-M(iddle) (containing RNA-2) and a relatively small amount of naturally occurring empty particles (CPMV-T(op)). The protein components of CPMV-B, CPMV-M, CPMV-T and a recombinantly-expressed empty VLP (eVLP-CPMV) are almost identical structurally (Fig. 1a)^13–16^ with the only significant difference being in the degree of cleavage of a 24 amino acid extension to the C-terminus of the S subunit, which occurs during particle purification and storage^13,14^. This extension is located at the 5-fold vertices of the eVLP-CPMV structure^13^. A polyclonal antiserum raised against WT CPMV^17^ can detect both the L and S subunits in all three forms of WT CPMV and eVLP-CPMV (Fig. 1b). An Affimer phage display library^3–5^ was screened against purified eVLP-CPMV immobilized using a biotin-streptavidin linkage, to isolate specific binders. After three rounds of panning, 24 randomly picked clones were tested for binding eVLP-CPMV as well as WT CPMV using phage ELISA (Fig. 1c). To confirm effective binding these Affimer clones were tested against WT CPMV. The majority of the clones bound WT CPMV to a level comparable to that seen for eVLP-CPMV. However, two clones (numbers 14 and 22), did not show such binding for CPMV or eVLP-CPMV and represent false positives from the screening process and were not analysed further (Fig. 1c). Seven clones with the highest signal amplification, and therefore most likely the highest affinity binders for CPMV and CPMV-eVLP (labelled with black asterisks (*), Fig. 1c), were sequenced. Sequence analysis revealed that all seven Affimers were unique. A prerequisite for our analysis of Affimer proteins was the selection of variable loops that had a significantly different sequence, since a similar sequence in this region suggests the binding sites are most likely identical. Three Affimer proteins (2, 11 and 24) were not taken forward for further analysis as the sequence in their variable loops was close to that of at least one of the other sequenced Affimer proteins. The remaining four Affimer proteins (3, 9, 17 and 23) were chosen for protein production (labelled with red asterisks (*), Fig. 1c). These were sub-cloned with a C-terminal His-Tag into pET11 expression vectors and subsequently produced in *E*. *coli* and purified for further analysis (see Methods and Supplementary Fig. 1a).

### Structure determination of CPMV bound to Affimer 3

To analyse the binding mechanism of CPMV to an Affimer reagent we chose to determine the structure of an Affimer:CPMV complex using cryoEM. Initially all four Affimer proteins were analysed using negative stain EM in complex with CPMV to find the Affimer reagent most suitable for cryoEM. Anti-CPMV Affimer 3 was selected as it did not cause appreciable aggregation or clumping of CPMV particles in negative stain screens. A negative stain dataset was collected and a preliminary 3D reconstruction was produced in RELION. However, the resolution was not sufficient to visualise the bound anti-CPMV Affimer 3, probably due to the size of the Affimer (11 KDa).

**Figure 2:**
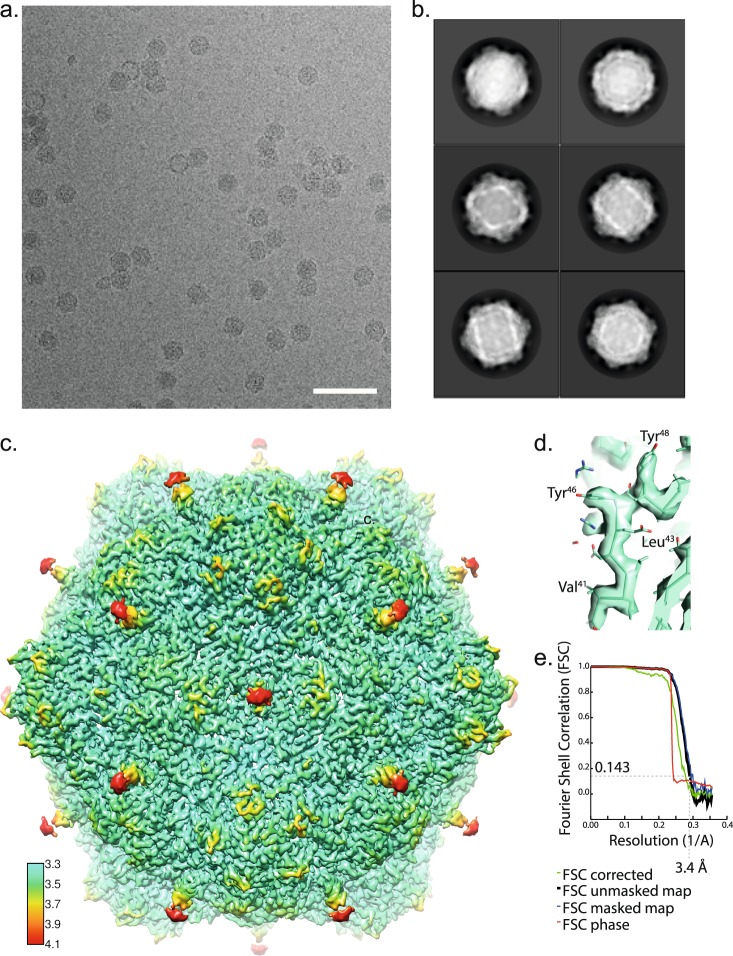
Cryo-Electron Microscopy of CPMV bound to anti-CPMV Affimer 3. (**a**) A representative cryoEM motion corrected micrograph of CPMV bound to anti-CPMV Affimer 3. Scale bar is 100 nm. (**b**) 2D class averages calculated in Relion1.4. (**c**) 3D cryo-EM reconstruction of CPMV bound to anti-CPMV Affimer 3 coloured by local resolution. The highest resolution region of the map is ~ 3.3 Å and is located at the viral capsid (blue/green). The lowest resolution features of the map (>4 Å) are the bound anti-CPMV Affimer proteins (red). A key is shown for reference. (**d**) Representative cryoEM density to demonstrate side chain density in the CP. (**e**) Fourier shell correlation (FSC) curve of the masked map, unmasked map and corrected map. The resolution reported here was according to the 0.143 criterion.

**Figure 3:**
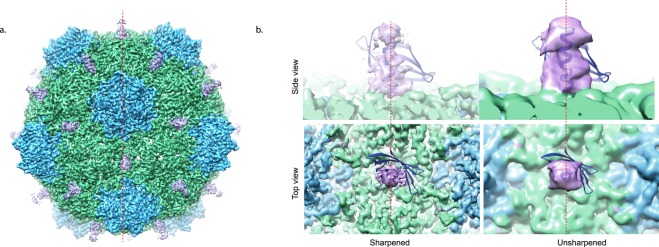
Anti-CPMV Affimer 3 binds to CPMV at the 2-fold axis. (**a**) 3D cryo-EM reconstruction of CPMV bound to anti-CPMV Affimer 3. The CPMV S subunit is coloured blue and the L subunit is green. Extra EM density attributed to anti-CPMV Affimer 3 is shown in purple. Icosahedral (I1) symmetry was imposed during image processing. The 2-fold axis is indicated with a dashed red line. (**b**) Zoomed in view of bound anti-CPMV Affimer 3 (purple) side and top views. The crystal structure of an Affimer (PDB 4N6U) is fitted into the density. As anti-CPMV Affimer 3 is bound to the 2-fold axis, density is not visualized for the beta strands due to averaging required for image processing. The sharpened map contains high resolution information and only the alpha helical portion of the Affimer can be visualized. The unsharpened map contains low resolution information where density for the beta strands can be visualized. The 2-fold axis is indicated with a dashed red line.

In an attempt to obtain a high resolution structure of the Affimer:CPMV complex, cryoEM grids were produced. Mixing CPMV with anti-CPMV Affimer 3 prior to loading onto a cryoEM grid and plunge freezing caused catastrophic aggregation, and the resulting grids were not suitable for high resolution data collection^18^. To overcome this issue CPMV was applied to lacey carbon grids overlaid with a thin (<3 nm) layer of carbon. This causes the CPMV particles to be immobilized on the carbon support, anti-CPMV Affimer 3 was subsequently applied, excess solution was removed and the grid was finally washed prior to blotting and plunge freezing (see Methods and^18^ for more details). This method resulted in high quality cryoEM grids with particles that were evenly distributed and thus ideal for data collection (Fig. 2a). Data were collected and an EM density map produced (see Methods). The global resolution of the resulting structure was 3.4 Å (Fig. 2, Table 1). The viral capsid has the highest resolution (Fig. 2c), and previous atomic models for the L and S subunit fit into the density with clear resolution of the bulky side-chains in both capsid proteins (Fig. 2d). The L subunits (green) interact at the 2-fold symmetry axis of the icosahedral capsid and here we can see additional density that is attributed to anti-CPMV Affimer 3 (purple, Fig. 3a). Due to the icosahedral averaging applied during image processing and structure determination, these locations have two-fold symmetry applied, which is appropriate for the viral CP, but not for the bound Affimer. As a result, the non-two-fold symmetric Affimer structure is inappropriately averaged meaning there is not clear density for the backbone of the Affimer and we are unable to map the position of individual amino acids. This is consistent with the Affimer protein being flexible relative to the virus CP as the resolution appears to be worse at the “tip” of the Affimer protein (Fig. 2c). There also appears to be a gap in density at the “tip” of the Affimer, presumably due to a high degree of flexibility. It is therefore difficult to interpret the resulting structure. We visualise the Affimer reagent bound to each of the twenty 2-fold symmetry axis. Due to symmetry averaging it is difficult to determine the occupancy of the Affimer reagent at these binding sites. The EM density attributed to the Affimer reagent is not as strong as the CP EM density but it is similar, therefore we believe the sites are substantially occupied. EM density is visualized for the alpha helical domain of the Affimer with the base of the helix bound at the interface between two L subunits (Fig. 3b). The Affimer’s beta strands are not visible at high resolution (Fig. 3b), suggesting that there is some flexibility in the binding of the Affimer. If we analyse the unsharpened EM density map which retains low-resolution information, the density corresponding to the β sheet is visible, but the strands are not resolved (Fig. 3b). The variable loop of the Affimer is the location for molecular recognition and therefore this region should be specifically bound to the capsid. Previous structural analysis of Affimer proteins has shown the location of the variable loop (Supplementary Fig. 1b). Due to the location of the variable loops, we propose that the N-terminus of the Affimer reagent is facing down into the capsid as this would allow the Affimer variable loops to bind to a portion of the L subunit in the CPMV capsid (Fig. 3b).

**Table 1:** Cryo-EM data collection and processing statistics for CPMV and anti-CPMV Affimer 3 cryo EM density.

	CPMV and anti-CPMV Affimer 3
**Data Collection**	
Micrographs	2,980
Pixel Size (Å/pixel)	1.4
Number of frames collected	35
Defocus Range (μm)	0.5–4.5
Voltage (kV)	300
Total Dose (e^−^/Å^2^)	45
**Data processing**	
Auto picking	144,258
2D classification	92,082
3D classification	15,441
Resolution	3.4 Å
Experimental B-factor (Å^2^)	−203
EMDB Accession number	EMD-4610

### Affimer 3 specifically binds to CPMV

**Figure 4:**
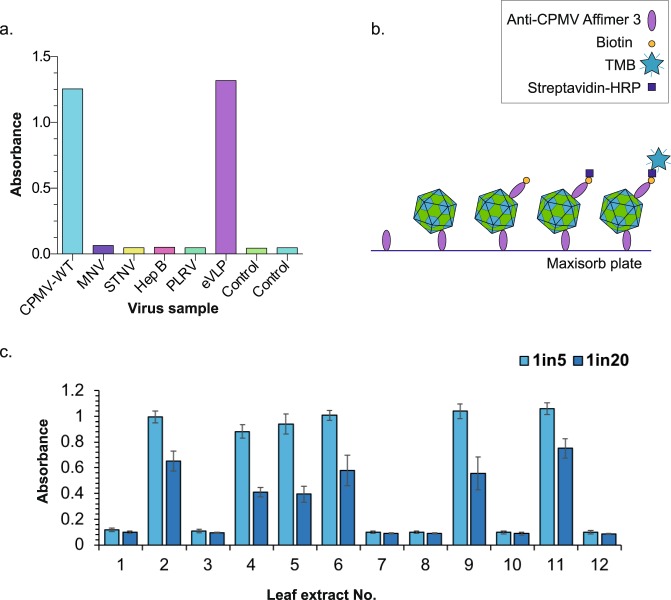
Anti-CPMV Affimer 3 is specific to CPMV. (**a**) ELISA of immobilized VLPs - Murine Norovirus (MNV), Satellite Tobacco Necrosis Virus (STNV), Hepatitis B (Hep B) and Potato Leaf Roll Virus (PLRV) detection using anti-CPMV Affimer 3. Only CPMV and eVLP-CPMV were detected by anti-CPMV Affimer 3. (**b**) A schematic describing a sandwich ELISA. Anti-CPMV Affimer 3 is immobilized on a maxisorb plate, if CPMV is present it binds to anti-CPMV Affimer 3. Biotinylated anti-CPMV Affimer 3 binds to the CPMV capsid (if present) which then binds to streptavidin-HRP. HRP oxidises TMB to produce a colour change which is monitored by absorbance. (**c**) A sandwich ELISA of a blind test to detect the presence of CPMV in infected leaves. 6 leaves were a negative control and 6 leaves were infected with CPMV (diluted to either 1 in 5 or 1 in 20). Leaf extract numbers 2, 4, 5, 6, 9 and 11 were positive. These crude leaf extracts also showed increased absorbance demonstrating the detection method was successful. Error bars show standard deviation from the mean.

For any binding reagent to be useful in diagnosis, it is essential that it does not produce false positive results. Therefore, we investigated whether the anti-CPMV Affimer 3 was specific for CPMV, or whether it would recognize the similar structural features of other icosahedral viruses. We therefore immobilized equal concentrations (10 µg/ml) of samples of the following VLPs; Murine Norovirus (MNV), Satellite Tobacco Necrosis Virus (STNV), Hepatitis B (Hep B) and Potato Leaf Roll Virus (PLRV) using a biotin-streptavidin linkage, and analyzed the ability of the anti-CPMV Affimer 3 to recognize them using an ELISA (Fig. 4a). Anti-CPMV Affimer 3 did not bind to any of the viruses tested but showed specificity for CPMV (both WT and eVLP).

To further test the suitability of anti-CPMV Affimer 3 for future in-field diagnostics, we tested the ability of anti-CPMV Affimer 3 to detect CPMV in crude extracts from infected leaves using a sandwich ELISA (Fig. 4b). In the field if a plant was found to be infected with CPMV (or another plant virus) the plant would be destroyed to limit the spread of the infection to other plants. Purified CPMV was used as a positive control to optimize the ELISA conditions. A sandwich ELISA requires two binding reagents, in this case two Affimer reagents; a “capture Affimer” which is immobilized on a plate and a “detection Affimer” which will bind to CPMV and produce a colour change via a Biotin-Streptavadin-HRP complex on addition of TMB (if CPMV is present) (Fig. 4b). The “detection Affimer” is cysteine terminated which permits conjugation to a biotin linker. The biotin is used for detection of a positive result in a sandwich ELISA as the cysteine linked “detection Affimer” will bind to CPMV if present. The “detection Affimer” is then able to bind to Streptavidin bound to HRP which will produce a colour change in TMB that can be monitored using absorbance in a plate reader (Fig. 4b). The “capture Affimer” does not require biotin conjugation and therefore Affimer proteins are usually produced without cysteines. This means Affimer reagents are easier to produce and the lack of a cysteine is useful in many downstream experiments. To find the optimal parameters for the type of capture anti-CPMV Affimer 3 (i.e. cysteine-terminated or “normal” (non-cysteine) Affimer), the concentration and incubation time of anti-CPMV Affimer 3 were determined (Supplementary Fig. 2a). When used as “capture” reagent, the cysteine-terminated anti-CPMV Affimer 3 displayed increased background compared to the non-cysteine anti-CPMV Affimer 3 (*, Supplementary Fig. 2a), therefore, the non-cysteine anti-CPMV Affimer 3 was used as the “capture Affimer” in subsequent ELISAs. The greatest absorbance was achieved with overnight adsorption at 4 °C of 50 µg/ml anti-CPMV Affimer 3 and with 0.5 µg/ml biotinylated cysteine-terminated detection anti-CPMV Affimer 3 (green box, Supplementary Fig. 2). Therefore, for the “capture Affimer”, 50 µg/ml of cysteine free “normal” Affimer reagent was incubated overnight at 4 °C and 0.5 µg/ml biotinylated cysteine terminated Affimer reagent was used as “detection Affimer”. These conditions were used in all the following ELISAs. To extract the CPMV particles from infected leaves two methods were compared (Supplementary Fig. 2b and see methods). We intentionally kept the preparation of leaves as simple as possible without the requirement for scientific equipment. Upon analysis of the most dilute leaf extractions, the highest absorbance was achieved by manually grinding the leaves in PBS buffer and so this method was used in subsequent analysis.

Blind testing was performed in which twelve leaves were randomly numbered 1–12 (6 of which were inoculated with CPMV, and 6 of which were uninoculated negative controls in a blind experiment). The leaves were extracted and analysed using the optimised sandwich ELISA conditions described above (Supplementary Fig. 2a). This extraction and sandwich ELISA was repeated three times, using a fresh sample of the numbered leaf each time (Fig. 4c). The ELISA correctly identified the CPMV-infected samples, with significant absorbance being observed from inoculated leaves (leaves 2, 4, 5, 6, 9, 11) and little absorbance (i.e. below a threshold of 0.2 optical density) was observed for the negative controls (leaves 1, 3, 7, 8, 10, 12) demonstrating that anti-CPMV Affimer 3 can specifically detect CPMV infected leaves (Fig. 4c).

## Discussion

There is a requirement for more robust and sensitive technologies for the detection of plant virus infections, as accurate detection is critical for farmers to implement control strategies and prevent disease outbreaks^19,20^. Current methods for detection are almost exclusively antibody-based systems such as ELISAs or similar technologies^2^. Such methods are often used in lateral flow devices which allow the detection of antigens in crude samples via a colour reaction; such devices are suitable for in-field deployment. Due to difficulties in production of monoclonal antibodies the majority of available plant virus antibodies used for diagnostic tests are polyclonal^21,22^, increasing the likelihood of false positive results. The use of antibodies has a number of disadvantages due to the expense of production but, more importantly, antibodies are not available for a number of important plant viruses. This is primarily due to the difficulty in production of WT viruses to high concentrations and sufficient purity but also due to the biocontainment issues involved with propagating WT viruses. Other techniques for plant virus detection include nucleic acid extraction and analysis^23^. This is usually based on polymerase chain reaction (PCR) followed by detection to analyze the genetic content of the virus^24–26^. The use of PCR for detection requires DNA sequencing therefore specialized personnel are required and samples must be sent to a laboratory. Due to the complex instrumentation and time-consuming nature of these techniques there is a strong interest in developing new systems for detection of plant pathogens^2^. There are a large number of research groups tackling this need and a number of commercial kits are available.

To overcome the disadvantages outlined above, we have demonstrated eVLPs can be successfully used to isolate Affimer reagents. Using a synthetic binder, such as an Affimer reagent, has a number of benefits compared with classic antibodies. For example, the most efficient and selective binders can be selected. This reduces the possibility of false positive results which is a common occurrence when using polyclonal antiserum. Additionally, Affimer reagents are cheap and relatively simple to produce. The use of VLPs as opposed to WT virus is also advantageous owing to the ease with which large quantities of VLPs can potentially be produced. In addition, isolation of eVLPs does not require live virus production therefore negating any biocontainment issues.

The results presented here demonstrate that we have identified an Affimer reagent which binds to WT and recombinant CPMV (Fig. 1). We have analysed the structure of CPMV in complex with the Affimer reagent using cryoEM (Fig. 2) revealing the binding site of the Affimer at the 2-fold symmetry axis of the viral capsid to the L subunit (Fig. 3). Finally, we have demonstrated that the Affimer reagent specifically detects CPMV in crude extracts of infected plant leaves (Fig. 4). Thus, the fundamental building blocks for a diagnostic test are in place, and could be applicable to a wide variety of plant virus infections and permit better management of crop viral infections in the field.

## Materials and Methods

### CPMV and CPMV eVLP purification

CPMV and CPMV eVLPs were produced as described in^13,14^.

### Western blot analysis

CPMV samples were boiled in SDS-PAGE sample buffer (240 mM Tris pH 6.8, 8% SDS, 40% glycerol, 0.1% bromophenol blue and 6.8% β-mercaptoethanol). Samples were separated by 8% SDS-PAGE, transferred to nitrocellulose, blocked with 1× PBS, 10% dried milk, 0.1% Tween 20, and probed with anti-CPMV at 1/2000^17^. The secondary antibody, anti-rabbit HRP (ab6721, Abcam), was used at 1/10 000. Blots were developed using enhanced chemiluminescence (ECL) solution (Amersham).

### Target preparation and phage display

Phage display screening was performed as previously described^3^.

### Phage ELISA

Phage ELISA screening was performed, as previously described, on 24 randomly selected clones from the final pan round^3^.

### Cryo-EM grid preparation and imaging

Ultrathin carbon film on lacey carbon support film 400 mesh grids (Agar Scientific) were glow discharged for 30 seconds (Pelco easiGlow). The grids were prepared using a Leica GP freezing device at 8 °C and 95% relative humidity by placing 3 μl of 0.1 mg/ml CPMV on to the grid and incubating for 30 seconds. Excess liquid was manually blotted prior to placing 3 μl of 0.01 mg/ml anti-CPMV Affimer 3 onto the grid and incubating for 30 seconds. Excess liquid was manually blotted and 3 μl of 100 mM sodium phosphate buffer, pH 7.0 was placed on the grid to wash away any unbound anti-CPMV Affimer 3. The grid was blotted automatically using the Leica GP freezing device prior to plunge freezing in liquid ethane cooled by liquid nitrogen. Data was collected on a FEI Titan Krios (eBIC, Harwell) transmission electron microscope at 300 kV, using an electron dose of **~**45 e^−^/Å^2^. The final object sampling was 1.4 Å per pixel. A total of 2,980 exposures were recorded using the EPU automated acquisition software on a 17 Hz FEI Falcon II direct electron detector. Each exposure movie had a total exposure of 2 seconds and contained 35 images.

### Image processing

Drift-corrected averages of each movie were created using MOTIONCORR^27^ and the contrast transfer function of each determined using gCTF^28^; any images showing signs of astigmatism were discarded. Approximately 1,000 particles were manually picked and classified using reference-free 2D classification in Relion (v1.4)^29^. The resulting 2D class average views were used as templates for automated particle picking using gAutomatch^30^ (see Table 1 for particle numbers at each processing step). The following processing was implemented using Relion(v1.4). To produce a structurally homogeneous subset of particles, the initial stack was reduced using a statistical particle sorting algorithm^31^, which excludes the particles that are least similar to the initial search references. The remaining particles were classified using reference-free 2D classification to yield a data set for 3D structure refinement that only includes isolated molecular views of CPMV (see Table 1).

We then searched within each data set for a subset of particle images with greater homogeneity and/or higher resolution, using sequential 3D classification steps with icosahedral symmetry imposed. Each of these steps split the data into two, and the class with the sharpest features and highest resolution was taken forward. The initial starting model was CPMV structure (EMDB-3013) filtered to 60 Å. To correct for mechanical drift, beam-induced movement and radiation damage, statistical movie processing and particle polishing procedures were implemented^29^. As CPMV particles are readily visible even in individual movie frames, a running average of three frames was used in the calculations. Frames 2–20 were included in the final reconstruction, the first frames were removed as these contain a large amount of movement from the beam initially hitting the sample, the final frames were discarded as these may contain radiation damage. Post-processing was employed to appropriately mask the model, estimate and correct for the B-factor of the maps^32^. The final resolution was determined using the ‘gold standard’ Fourier shell correlation (FSC.0.143) criterion^33^ as 3.44 Å (FSC curves are shown in Fig. 2e). Local resolution was estimated using the local resolution feature in RELION2.0^34^.

### Affimer protein production and biotinylation

Anti-CPMV Affimer 3 was produced and labelled with biotin as previously described^4^.

### ELISA analysis with purified viruses

Maxisorb plates (Nunc) were coated with virus samples (10 µg/ml) overnight at 4 °C and then blocked with 2x casein blocking buffer (Sigma) overnight at 37 °C. The plates were washed once and incubated with 5 µg/ml biotinylated anti-CPMV Affimer 3 for 1 h at room temperature. Subsequently the plate was washed once and bound anti-CPMV Affimer 3 were detected by a 1:1000 dilution of HRP-conjugated streptavidin (Pierce) for 1 h at room temperature. Following 12 times washing Affimer 3 binding was visualised with 3,3′,5,5′ -tetramethylbenzidine (TMB) (Seramun) and measured at 610 nm.

### Preparation of CPMV infected leaves

CPMV-infected *N*. *benthamiana* leaves were produced by agroinfiltration of 3-week-old plants with pNinP-S1NT^35^ and pEAQ-RNA-2^13^. Infiltrated leaves were harvested 7 days post infection. The uninfected negative control was grown alongside. Leaves were frozen at −20 °C prior to analysis.

### Sandwich ELISA

Maxisorb plates (Nunc) were coated with anti-CPMV Affimer 3 (concentration and incubation time as shown in Supplementary Fig. 2a) at 4 °C, washed three times and then blocked with 1x casein blocking buffer (Sigma) for 1 hour at room temperature. The plates were washed three times and incubated with 25 µl purified CPMV or leaf extract (diluted in casein blocking buffer to the concentration shown in Supplementary Fig. 2b and Fig. 4) for 1 hour at room temperature. Subsequently the plate was washed three times and incubated with 25 µl of biotinylated cysteine-terminated detection anti-CPMV Affimer 3 (diluted in casein blocking buffer to the concentration shown in Supplementary Fig. 2a) for 1 hour at room temperature, the plates were subsequently washed three times. Bound CPMV and anti-CPMV Affimer were detected by a 1:1000 dilution of HRP-conjugated streptavidin (Pierce) for 1 h at room temperature. The plates were washed six times prior to analysing the binding of CPMV and anti-CPMV Affimer 3 using TMB (Seramun) absorbance at 650 nm.

The optimal sandwich ELISA conditions were determined as; capture anti-CPMV Affimer 3 at 50 µg/ml overnight at 4 °C and detection anti-CPMV Affimer 3 at 0.5 µg/ml.

### Leaf sample preparation optimisation

CPMV was extracted from leaf samples using two methods. The first method of extraction was to grind 8 mg of leaf in 80 μL of PBS containing complete EDTA-free protease inhibitor cocktail (at the manufactures recommended concentration) for five minutes, prior to spinning down at 17000 *g* for five minutes to remove debris. The second method was to use a commercial kit (P-PER plant protein extraction kit, Thermofisher) for which 80 mg of leaf is extracted into 800 μL solution.

The concentration of CPMV in infected leaves was estimated using a calibration curve generated using purified CPMV (Supplementary Fig. 2c). The CPMV concentrations in each extracted leaf sample were calculated (Supplementary Fig. 2d), as there was 100 mg of leaf per ml, 50 μg/ml CPMV in the extracted solution is equivalent to 0.5 μg extracted CPMV per mg of leaf.

## Data Availability

Coordinates of four CPMV subunits with the bound Affimer rigid body fitted have been deposited in the Protein Data Bank under accession code 6QOZ. The cryoEM reconstruction is deposited in the EM Data Bank under accession codes EMD-4610. All reagents and relevant data are available from the authors upon request.
